# MDISCO: A High-Throughput Tissue-Clearing Protocol for Preservation of Endogenous Fluorescence in Whole Mouse Brains

**DOI:** 10.21769/BioProtoc.5649

**Published:** 2026-04-05

**Authors:** Madeline Martinez, Jake Thornberry, Akihiko Ozawa, Lawrence Toll

**Affiliations:** 1 Department of Biomedical Science, Charles E. Schmidt College of Medicine, Florida Atlantic University, 5353 Parkside Drive, Jupiter, FL, USA; 2 Stiles-Nicholson Brain Institute, Florida Atlantic University, Jupiter, FL, USA; 3 Harriet l. Wilkes Honors College, Florida Atlantic University, Jupiter, FL, USA

**Keywords:** Tissue clearing, Whole-brain imaging, Solvent-based clearing, Light-sheet microscopy, MDISCO

## Abstract

Organic solvent–based tissue clearing methods are widely used for whole-brain imaging but often compromise endogenous fluorescence. Existing protocols, such as iDISCO and fluorescence-preserving variants, have improved optical transparency but still present trade-offs between fluorescence retention, tissue stability, and workflow complexity. Here, we present MDISCO, a modified iDISCO-based clearing protocol designed to enhance preservation of endogenous fluorescence while maintaining high transparency and stable tissue morphology. MDISCO is directly compared with FDISCO+, an established fluorescence-preserving protocol, for the preservation of endogenous tdTomato and YFP. Performance across clearing steps is evaluated by measuring brain weight, anteroposterior and mediolateral dimensions, and optical transparency before and after solvent clearing and refractive index matching. Fluorescence preservation is assessed using whole-brain light-sheet microscopy with standardized imaging parameters to enable direct comparison. This protocol provides an accessible and high-throughput, reproducible workflow for solvent-based clearing with robust endogenous fluorescence preservation, offering clear advantages for whole-brain 3D imaging of genetically encoded fluorescent reporters.

Key features

• Preserves endogenous tdTomato and YFP fluorescence in whole mouse brains without signal amplification through immunolabeling.

• Improves optical clarity and cellular resolvability while maintaining anatomical integrity.

• Supports high-throughput “clearing” of whole-tissue samples.

## Graphical overview


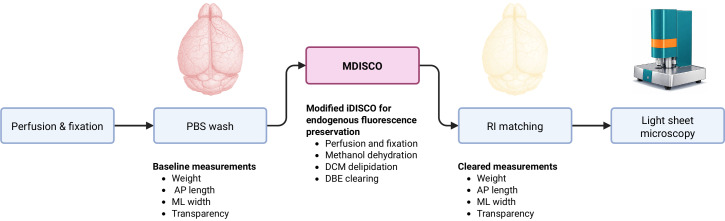


**Experimental overview of MDISCO.** Whole mouse brains are perfused, fixed, and stored in PBS prior to baseline measurements of weight, anteroposterior (AP) length, mediolateral (ML) width, and transparency. Samples are then processed using MDISCO via dichloromethane (DCM) delipidation and dibenzyl ether (DBE) clearing. Following refractive index (RI) matching, post-clearing measurements are collected, and brains are imaged by light-sheet microscopy.

## Background

Tissue-clearing methods have enabled high-resolution imaging of intact biological samples by rendering tissue optically transparent while preserving structural organization. In rodent brain imaging, these approaches facilitate visualization of genetically encoded fluorescent reporters and large-scale neural circuits without the need for physical sectioning [1,2]. However, many organic solvent–based clearing protocols result in substantial quenching of endogenous fluorescent proteins, often necessitating additional immunolabeling steps that increase experimental complexity, cost, and processing time [2,3].

Organic solvent–based tissue clearing was first introduced with the 3DISCO protocol, which achieves optical transparency through graded dehydration in tetrahydrofuran (THF), followed by delipidation with dichloromethane (DCM) and refractive index matching in dibenzyl ether (DBE) [3]. This approach enables rapid and effective clearing of whole organs but is associated with substantial quenching of endogenous fluorescent proteins, limiting its utility for experiments relying on genetically encoded reporters [2,3]. The iDISCO protocol adapted this framework to support whole-mount immunolabeling by replacing THF with methanol-based dehydration, introducing hydrogen peroxide bleaching to reduce background, and incorporating rehydration and permeabilization steps to facilitate antibody penetration [3]. While iDISCO greatly expanded the applicability of solvent-based clearing for molecular labeling, it was not specifically optimized to preserve endogenous fluorescence and often results in diminished fluorescent protein signal in cleared tissue.

Several optimized clearing strategies, including uDISCO (ultimate DISCO) and FDISCO (fluorescence-preserving DISCO)-based methods, have been developed to improve fluorescence retention relative to earlier protocols [4,5]. While some of these approaches reduce regulatory and handling constraints by avoiding certain organic solvents, practical challenges related to incomplete clearing, background haze, and prolonged refractive index matching times can still limit imaging efficiency and data quality. In particular, residual scattering and bubble formation within incompletely cleared tissue can substantially increase imaging time and reduce effective cellular resolvability, even when protocols are followed precisely.

Immunolabeling is frequently used to visualize molecular targets in cleared tissue; however, this approach can introduce substantial variability due to incomplete antibody penetration, nonuniform labeling, off-target binding, and increased background signal, particularly in large or densely packed samples. Antibody-based labeling also substantially extends processing time and increases experimental cost, and labeling efficiency can vary across brain regions and experimental batches. In experimental contexts where genetically encoded fluorescent reporters are already present, preserving endogenous fluorescence avoids the additional cost, time, and variability associated with antibody-based signal amplification, including antibody optimization, staining condition adjustments, and reagent selection. While immunolabeling remains essential for targets that cannot be genetically encoded, these limitations motivate the continued development of clearing approaches that preserve endogenous fluorescence when reporter expression is available.

To address these limitations, we developed MDISCO (modified DISCO), a modified iDISCO (immunolabeling-enabled DISCO)-based clearing protocol designed to preserve endogenous fluorescence while maintaining optical clarity and anatomical integrity. Targeted adjustments to solvent handling and exposure conditions were implemented to minimize fluorophore quenching and improve clearing uniformity. These modifications enable consistent, high-quality whole-brain imaging without reliance on antibody labeling or proprietary reagents.

Subsequent DISCO-derived protocols have sought to balance optical transparency, tissue integrity, and preservation of endogenous fluorescence. uDISCO introduced solvent-mediated tissue shrinkage to improve imaging depth and resolution, but retained aggressive dehydration conditions that can compromise fluorescent protein stability [4]. More recent fluorescence-preserving approaches, such as FDISCO and FDISCO+, employ THF-based dehydration in combination with antioxidant additives to reduce fluorophore quenching, achieving improved fluorescence retention at the cost of increased workflow sensitivity and prolonged refractive index matching [5,6].

Building on these prior methods, MDISCO is derived from the iDISCO framework and retains methanol-based dehydration and peroxide bleaching while introducing targeted modifications to solvent exposure timing and post-clearing handling. In MDISCO, graded methanol dehydration is used in place of THF to reduce exposure to highly aggressive organic solvents known to destabilize fluorescent protein chromophores. DCM exposure is limited to short, defined delipidation steps rather than prolonged clearing, minimizing cumulative solvent-induced fluorescence loss while still achieving effective lipid removal. Equilibration in dibenzyl ether (DBE) is used primarily for uniform refractive index matching and to facilitate air bubble dissipation, followed by transfer to ethyl cinnamate (ECi) for long-term storage and imaging. ECi provides stable refractive index matching and has been reported to exhibit improved compatibility with endogenous fluorescent proteins. Together, these targeted modifications are designed to preserve fluorescent protein signal while maintaining optical clarity and tissue morphology, resulting in a practical and reproducible workflow that reduces variability and improves imaging readiness for whole-brain fluorescence microscopy.

In this protocol, MDISCO is evaluated in direct comparison with FDISCO+ using whole brains from tdTomato- and YFP-expressing transgenic mice. Tissue morphology, optical transparency, and endogenous fluorescence preservation are assessed across defined stages of the clearing workflow. Together, these analyses provide a practical framework for evaluating fluorescence-preserving tissue-clearing methods based not only on chemical composition but also on imaging performance, data interpretability, and experimental efficiency.

## Materials and reagents

**Biological materials**

1. B6.Cg-*Gt(ROSA)26Sor^tm9(CAG-tdTomato)Hze^*/J mice [Jackson Laboratory, strain #007909; common name: Ai9 or Ai9(RCL-tdT)] [7]

2. STOCK *Fos^tm2.1(icre/ERT2)Luo^*/J mice [Jackson Laboratory, strain #030323, common name: Fos^2A-iCreER^ (TRAP2)] [8]

3. TRAP2/Ai9 mice [TRAP2 (*Fos^2A-iCreER^*) and *Rosa26LSL-tdTomato* (Ai9) mice were crossed in-house mice] [9]

4. B6.129(Cg)-*Oprl1^tm1.1Mrbr^*/J mice (gifted from the Bruchas Lab at the University of Washington, Jackson Laboratory, strain #036308, common name: NOPR^loxP/YFP^) [10]

**Reagents**

1. Paraformaldehyde (PFA) powder, EM-grade (Fisher Scientific, catalog number: AC416785000)

2. PBS powder, pH 7.4 (Sigma-Aldrich, catalog number: P3813)

3. Methanol, ≥99.8% (Sigma-Aldrich, catalog number: 179957)

4. Dichloromethane (DCM), ≥99.9% (Applied Biosystems^TM^, catalog number: 402152)

5. Hydrogen peroxide (H_2_O_2_) solution, 30% (Thermo Scientific, catalog number: 033323.AY)

6. Dibenzyl ether (DBE), ≥99% (Thermo Scientific, catalog number: A18447.36)

7. Ethyl cinnamate (ECi), ≥98% (Thermo Scientific, catalog number: A12906.36)

8. Double-distilled H_2_O (laboratory supply)

**Laboratory supplies**

1. Solvent-resistant gloves

2. Glass scintillation vials (e.g., Grainger, catalog number: 3LDT2)

## Equipment

1. 37 °C incubator (e.g., Benchmark Scientific^TM^, SKU: RS7165)

2. Nutating mixer (Fisher Scientific, catalog number: 88-861-041)

3. Light-sheet microscope (e.g., UltraMicroscope Blaze, Miltenyi Biotec)

## Software and datasets

1. ImSpector Pro (Miltenyi Biotec, version 8.0.3, requires a license)

2. MACS iQ View, 3D Large Volume Software (Miltenyi Biotec, version 1.2.3, requires a license)

3. Imaris (Oxford Instruments, Version 11.0, requires a license)

4. FIJI (ImageJ)

## Procedure

**Day 1. Perfusion and fixation**

1. Anesthetize the mouse according to approved institutional animal care protocols.

2. Perfuse transcardially with 20–30 mL of 1× PBS at a flow rate of approximately 5–10 mL/min until the liver clears.

3. Immediately perfuse with 20–30 mL of freshly prepared 4% (w/v) PFA in PBS at the same flow rate. PFA powder is dissolved in 1× PBS at 60 °C with 1 M NaOH added dropwise to facilitate dissolution; pH is adjusted to 7.4. The solution is cooled and filtered through a 0.45 μm filter before use.

4. Dissect the brain carefully and immerse it in 10–15 mL of 4% PFA to ensure complete submersion.

5. Fix overnight at 4 °C with gentle agitation if possible.

**Day 2. PBS washes (37 °C)**

6. Transfer the fixed brain into a clean container containing 1× PBS.

7. Incubate overnight at 37 °C with rocking if possible.

**Day 3. Methanol dehydration [room temperature (RT)]**

8. Inspect the sample and ensure all hair or debris is removed from the brain surface.

9. Transfer the brain sequentially through the following MeOH/water (v/v) gradients, each for 1 h at RT, enough to fill the vial of your choice completely: 20% MeOH; 40% MeOH; 60% MeOH; 80% MeOH.

*Note: All solvent concentrations and mixture ratios in this protocol are defined as (v/v) unless otherwise stated.*

10. Incubate the brain in 100% MeOH for 1 h at RT.

11. Replace with fresh 100% MeOH and incubate overnight at RT.

**Pause point:** The samples can be stored in 100% MeOH (see General note 3).

**Day 4. Delipidation/permeabilization (RT)**

12. Transfer the brain to 66% DCM/33% MeOH.

**Caution:** Many organic solvents soften many plastics; use only solvent-resistant containers.

13. Incubate overnight at RT with rocking.

**Day 5. Bleaching (4 °C)**

14. Wash the brain in 100% MeOH for 2 h at RT.

15. Replace with fresh 100% MeOH and incubate an additional 2 h at RT.

16. Prepare 6% H_2_O_2_ in MeOH fresh just before use (1 part 30% H_2_O_2_: 4 parts MeOH).

17. Incubate the brain in 6% H_2_O_2_/MeOH overnight at 4 °C.

**Critical:** Always prepare fresh 6% H_2_O_2_/MeOH for bleaching; old peroxide increases background.

**Day 6. Post-bleaching MeOH washes (RT)**

18. Wash the brain in 100% MeOH for 1 h at RT.

19. Replace with fresh 100% MeOH and incubate overnight at RT.

**Day 7. Final delipidation and clearing**

20. Incubate the brain in 66% DCM/33% MeOH for 3 h at RT.

21. Replace the solution with 100% DCM and incubate for 1 h at RT.

22. Transfer the brain to 100% DBE.

23. Rock at RT until the tissue becomes cleared (typically several hours).

**Final transfer to imaging medium (ECi)**

**Figure 1.**
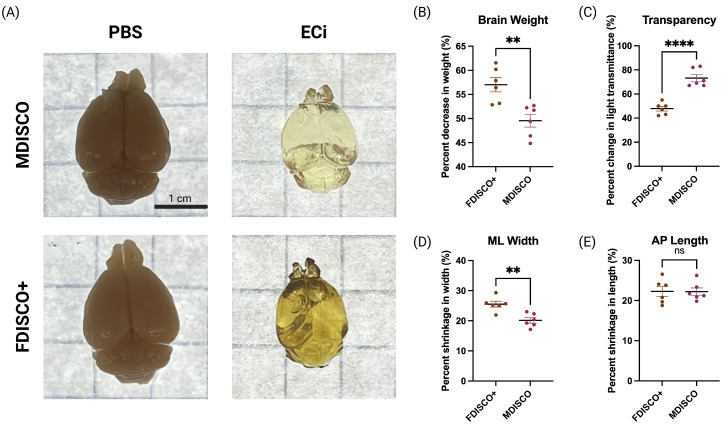
Brain morphology and transparency across processing stages. (A) Representative dorsal views of whole mouse brains at baseline (PBS) and after refractive index matching in ethyl cinnamate (ECi) following processing with MDISCO or FDISCO+. Scale bar, 1 cm. (B–E) Quantification of percent change relative to PBS baseline in brain weight (B), light transmittance (C), mediolateral (ML) width (D), and anteroposterior (AP) length (E) after clearing. Each dot represents an individual brain; horizontal lines indicate mean ± SEM. Statistical comparisons between MDISCO and FDISCO+ were performed using unpaired t tests with Welch’s correction. **p < 0.01, ****p < 0.0001; ns, not significant.

24. Keep the brain in DBE for 4–5 days, allowing internal bubbles to dissipate fully. Optional: Place in a 37 °C incubator, checking hourly, to accelerate bubble removal.

25. Once bubbles are eliminated and the tissue appears optically uniform, replace the solution with ECi for final RI matching and storage. Final morphological measurements are taken after this stage (Figure 1).

## Data analysis

Morphological measurements and fluorescence visualization were used to compare tissue stability, optical clarity, and endogenous fluorescence preservation between MDISCO and FDISCO+. All analyses were performed on six biologically independent mouse brains per clearing method. Whole-brain images were acquired on the Miltenyi UltraMicroscope Blaze II using ImSpector Pro acquisition software. Image tiles were stitched using Miltenyi Macs iQ software, and stitched volumes were visualized and sectioned using Imaris.

**Morphological measurements**

Brain weight, anteroposterior (AP) length, and mediolateral (ML) width were measured at two stages of processing: (1) post-fixation in PBS (baseline) and (2) after refractive index matching in ethyl cinnamate (ECi). AP and ML dimensions were measured in FIJI by calibrating image scale and using the straight-line tool to quantify AP length (rostral cerebrum to caudal cerebellum) and ML width (widest point of the cerebrum). Percent changes relative to the PBS baseline were calculated for each brain to assess tissue shrinkage or expansion across the clearing workflow.

**Transparency analysis**

Optical transparency was evaluated in PBS and after refractive index matching in ECi. Transparency was quantified from raw brightfield images using FIJI by calculating normalized transmittance, defined as the ratio of the mean grayscale intensity within a whole-brain region of interest (ROI) to the mean grayscale intensity of adjacent background paper over the same backlight within each image. This normalization controlled for variability in illumination conditions across imaging sessions.

**Step-by-step (FIJI)**

1. Open the image: *File* → *Open…*

2. Convert to 8-bit (for consistent intensity measurements across files): *Image* → *Type* → *8-bit* (If you want to preserve original bit depth, you can skip this, but be consistent across all samples.)

3. Set measurements: *Analyze* → *Set Measurements.* Check mean gray value and area. Click OK.

4. Define the “Brain ROI.” Select the Freehand selection tool (or Polygon tool). Carefully outline the entire brain, excluding obvious glare/specular highlights if present. Add the ROI to ROI Manager: *Analyze* → *Tools* → *ROI Manager* → *Add.*

5. Measure the brain intensity. With the brain ROI selected, *Analyze* → *Measure.* Record Mean as: I_brain.

6. Define the “Background ROI.” Choose a background region on the same image (e.g., adjacent white paper) that is close to the brain, is not shadowed, and does not include edges, text, glare, or objects. Use a rectangle (recommended for consistency) of the same approximate size across images. Add to *ROI Manager* → *Add.*

7. Measure background intensity: Select the background ROI → *Analyze* → *Measure.* Record Mean as: I_bg.

8. Calculate normalized transmittance.

Compute: Normalized transmittance (T) = I_brain/I_bg. Calculate as a percent:

% transparency = 100 × (I_brain/I_bg)

**Fluorescence preservation**

Endogenous fluorescence preservation was assessed using whole-brain light-sheet imaging with identical acquisition settings across samples. Imaging parameters were held constant within fluorophore groups to allow direct qualitative and quantitative comparison of signal intensity and spatial continuity between clearing methods. Brains exhibiting mechanical damage, incomplete clearing, or obvious imaging artifacts were excluded from analysis.

**Cellular resolvability (edge sharpness)**

To quantitatively assess preservation of resolvable cellular structure independent of bulk fluorescence intensity, an edge-based sharpness metric was calculated from two-dimensional coronal sections. For each brain, square regions of interest (ROIs) were manually selected within primary somatosensory cortex (SSp) and dorsal striatum (STRd) using anatomical landmarks and fully contained within gray-matter parenchyma, avoiding tissue edges, ventricles, and obvious artifacts. ROIs were extracted from coronal sections corresponding to a mid-striatal anatomical level, with identical ROI dimensions applied across all samples.

ROI images were exported at full bit depth without intensity rescaling and analyzed in FIJI/ImageJ. Edge sharpness was quantified by applying a Sobel edge-detection filter (*Process* → *Find Edges*), which highlights high-frequency spatial intensity gradients corresponding to cellular boundaries. The mean gray value of the resulting edge-filtered image was measured and used as an index of edge sharpness. Higher values indicate increased preservation of sharp cellular features and improved resolvability, whereas incompletely cleared or optically hazy tissue yields reduced edge signal despite potentially elevated raw fluorescence intensity. Edge sharpness measurements were analyzed separately for SSp and STRd. Each ROI contributed a single measurement per brain.

**Step-by-step (FIJI)**

1. Open the section image: *File* → *Open…*

2. Convert to 8-bit: *Image* → *Type* → *8-bit* (again, keep consistency across all samples).

3. Select a matched anatomical level: Use the same AP level across brains, and use landmarks (lateral ventricles, caudoputamen shape, corpus callosum) to match.

4. Set measurements: *Analyze* → *Set Measurements.* Check the mean gray value and area. Click OK.

5. Create a fixed-size ROI: Choose a square ROI size and use it for every sample (e.g., 300 × 300 μm or 512 × 512 pixels, depending on export resolution). You can enforce this by *Edit* → *Selection* → *Specify…* (enter width/height in pixels).

6. Place ROI in the target region: Place ROI fully within gray matter. Avoid tissue edges, ventricles, tears, bubbles, and obvious streaking. Do this for each ROI separately. Add each ROI to the ROI Manager for record-keeping.

7. Duplicate ROI into a new image (prevents accidental edits to original): With ROI selected, *Image* → *Duplicate…* Check “Duplicate selection” → OK. This creates a small ROI-only image.

8. Apply Sobel edge detection. On the ROI-only image, *Process* → *Find Edges* (FIJI applies a Sobel-like edge filter; this converts sharp transitions into a strong edge signal).

9. Measure mean edge intensity: *Analyze* → *Measure*.

**Record mean as EdgeSharpness**

10. Repeat for each ROI for each brain. Use the same ROI size, same workflow, and same export settings across all samples.

**Statistical analysis**

All quantitative measurements are reported as mean ± standard deviation. Statistical comparisons between clearing methods were performed using unpaired two-tailed Student’s *t*-tests, with significance defined as p < 0.05. Image processing and measurements were performed using FIJI/ImageJ. Whole-brain visualization and sectioning were conducted using Imaris. Data organization and statistical analyses were performed using standard spreadsheet software and/or R. Familiarity with image analysis software and basic statistical testing is required; no specialized computational infrastructure or command-line expertise is necessary.

## Validation of protocol

To validate the performance and reproducibility of the MDISCO clearing protocol, tissue morphology, optical transparency, and endogenous fluorescence preservation were quantitatively compared with FDISCO+ [6]. As shown in Figure 1, both clearing methods produced measurable changes in brain morphology relative to the PBS baseline. MDISCO- and FDISCO+-cleared brains exhibited comparable changes in anteroposterior (AP) length, with no statistically significant difference between methods. In contrast, mediolateral (ML) shrinkage was significantly reduced in MDISCO-cleared samples relative to FDISCO+, indicating improved preservation of lateral tissue dimensions. Brain weight changes followed a similar trend, with MDISCO showing reduced variability across samples. Consistent with prior reports indicating that peroxide formation in DBE at room temperature contributes to endogenous fluorescence decay, prolonged exposure to DBE was avoided in this protocol [6]. Optical transparency, quantified as normalized light transmittance, was significantly increased in MDISCO-cleared brains compared to FDISCO+, reflecting more uniform refractive index matching and reduced residual scattering. Although the FDISCO+ protocol was implemented repeatedly with careful pH control, recommended antioxidant additives, and early transfer to ECi, its performance was variable and consistently inferior to MDISCO under our experimental conditions, highlighting the sensitivity of THF-based workflows to experimental context.

Fluorescence preservation was evaluated to assess the robustness and reproducibility of the MDISCO clearing protocol. All images were acquired using a Miltenyi UltraMicroscope Blaze II light-sheet microscope with identical acquisition settings applied across samples within each fluorophore group to control for imaging-related variability. Imaging was performed using 60% laser power, a step size of 3 μm, a light-sheet numerical aperture (NA) of 3.9 μm, and a sheet width set to 100%. Images were collected in light speed mode using fast tiling scan with a scaling factor of 3.0, an exposure time of 25 ms, a 4× objective, and 1× zoom, with samples immersed in ethyl cinnamate (ECi) during imaging. Whole-brain datasets were stitched using MACS iQ View with default stitching parameters, as the UltraMicroscope Blaze II and MACS iQ View software are designed to interface directly within the Miltenyi imaging ecosystem.

Stitched volumes were imported into Imaris and visualized in *Volume* view. Orthogonal sections were generated using the *Orthogonal Slicer* tool with a slice thickness of 40 μm to match the thickness commonly used in traditional sectioned histology.

**Figure 2.**
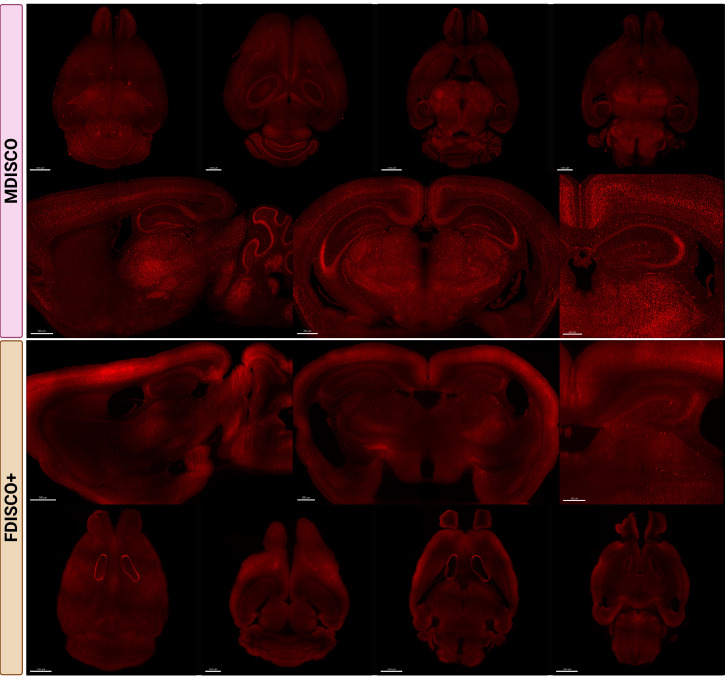
MDISCO preserves cellular resolvability and uniform tdTomato fluorescence throughout the mouse brain compared to FDISCO+. Representative whole-brain and sectional images from TRAP2/Ai9 mouse brains cleared using MDISCO (top) or FDISCO+ (bottom). In TRAP2/Ai9 mice, tdTomato expression is expected to be broadly distributed across brain regions, reflecting activity-dependent labeling of neurons activated during the defined TRAP window. All samples were imaged on the Miltenyi UltraMicroscope Blaze II using identical acquisition settings within the fluorophore group. Representative FDISCO+-cleared sample showing non-removable, persistent internal air bubbles despite 1-week incubation in dibenzyl ether (DBE) prior to ethyl cinnamate (ECi) transfer. Top rows show dorsal and ventral whole-brain views, while lower rows show representative sagittal and coronal sections at matched anatomical levels. Scale bars: 1500, 1000, 1000, and 1500 µm (MDISCO coronal overview, from left to right); 500, 700, and 500 µm (MDISCO higher-magnification panels, from left to right); 500 µm (FDISCO+ coronal overview panels); 1500, 1000, 1500, and 1500 µm (FDISCO+ dorsal overview panels, from left to right).

**Figure 3.**
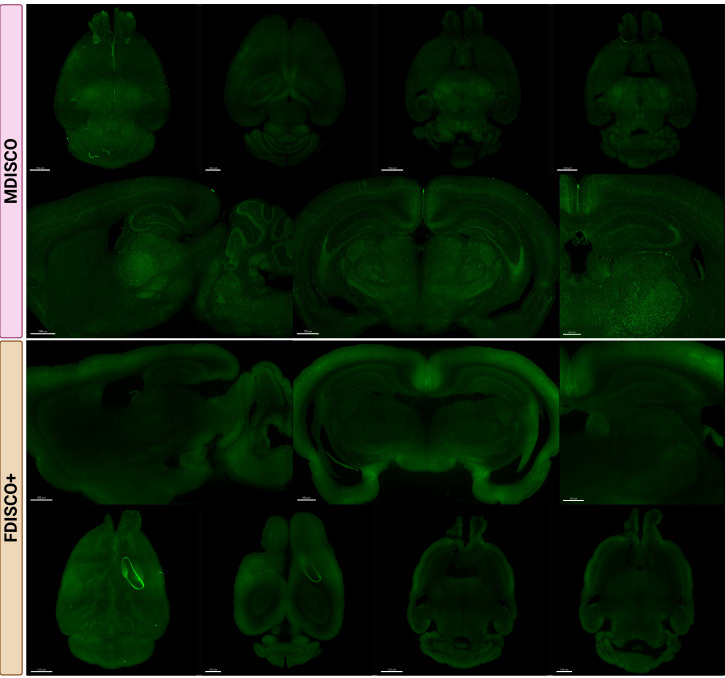
MDISCO preserves cellular resolvability and uniform YFP fluorescence throughout the mouse brain compared to FDISCO+. Representative whole-brain and sectional images from NOPR^loxP/YFP^ mouse brains cleared using MDISCO (top) or FDISCO+ (bottom). In NOPRloxP/YFP mice, YFP expression is expected to be broadly distributed throughout the brain, consistent with widespread expression of the nociceptin/orphanin FQ (NOP) receptor. All samples were imaged on the Miltenyi UltraMicroscope Blaze II using identical acquisition settings within the fluorophore group. Representative FDISCO+-cleared sample showing non-removable, persistent internal air bubble on the right side despite 1-week incubation in dibenzyl ether (DBE) prior to ethyl cinnamate (ECi) transfer. Top rows show dorsal and ventral whole-brain views, while lower rows show representative sagittal and coronal sections at matched anatomical levels. Scale bars: 1500, 1000, 1500, and 1500 µm (MDISCO coronal overview panels, from left to right); 1000, 700, and 500 µm (MDISCO higher-magnification panels, from left to right); 800, 500, and 500 µm (FDISCO+ sagittal overview panels, from left to right); 1500, 1000, 1500, and 1000 µm (FDISCO+ dorsal overview panels, from left to right).

Endogenous fluorescence preservation was examined in tdTomato-expressing (Figure 2) and YFP-expressing (Figure 3) mouse brains following clearing. After one week of RI matching, MDISCO-cleared samples showed no visible internal air bubbles, whereas FDISCO+-cleared samples almost always retained persistent bubbles that interfered with imaging. For each fluorophore and clearing method, six biologically independent brains were processed and analyzed as described in the Data Analysis section. Representative images are shown from two independent samples per strain and protocol to illustrate reproducibility across biological replicates. For each condition, one sample is presented as a whole-brain view with representative imaging depths, while a second sample is shown with matched sagittal and coronal sections and higher-magnification regions of interest.

**Figure 4.**
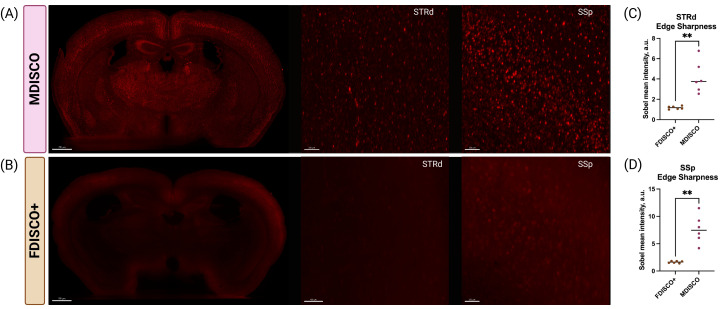
MDISCO preserves cellular resolvability compared to FDISCO+ as quantified by edge-based sharpness analysis. (A–B) Representative coronal sections from tdTomato-expressing mouse brains cleared using MDISCO (A) or FDISCO+ (B) and imaged under identical acquisition settings. Whole-section views are shown alongside higher-magnification regions of interest (ROIs) fully contained within the dorsal striatum (STRd) and primary somatosensory cortex (SSp). MDISCO-cleared tissue exhibits clearly defined cellular features and reduced background haze, whereas FDISCO+-cleared tissue displays elevated diffuse signal with diminished structural definition. (C–D) Quantification of cellular resolvability using an edge-based sharpness metric in STRd (C) and SSp (D). Edge sharpness was calculated as the mean intensity following Sobel edge detection of 2D ROI images in FIJI. Each point represents one biologically independent brain (n = 6 per clearing method), with horizontal bars indicating mean ± SEM. MDISCO-cleared samples exhibited significantly higher edge sharpness in both regions compared to FDISCO+, indicating improved preservation of resolvable cellular structure. p < 0.01 (unpaired two-tailed Student’s t-test). Scale bars: 700, 300, and 300 µm (MDISCO panels, from left to right); 700, 400, and 300 µm (FDISCO+ panels, from left to right).

To quantitatively assess preservation of interpretable cellular structure independent of bulk fluorescence intensity, cellular resolvability was evaluated using an edge-based sharpness metric (Figure 4). Sobel edge detection revealed significantly higher edge sharpness values in MDISCO-cleared brains compared to FDISCO+ in both dorsal striatum (STRd) and primary somatosensory cortex (SSp), indicating improved preservation of high-frequency spatial features corresponding to cellular boundaries. In contrast, FDISCO+-cleared samples exhibited low edge sharpness despite occasional increases in overall fluorescence intensity, consistent with elevated background haze and reduced structural definition observed qualitatively.

Together with morphological and transparency measurements (Figure 1), these results demonstrate that while anteroposterior (AP) shrinkage did not differ significantly between clearing methods, MDISCO produced reduced mediolateral (ML) shrinkage, increased optical transparency, and significantly improved cellular resolvability across multiple brain regions. These quantitative and qualitative findings, supported by analyses described in the Data analysis section, establish that MDISCO provides robust and reproducible preservation of endogenous fluorescent protein signal that is both brighter and more structurally interpretable than FDISCO+.

## General notes and troubleshooting

**General notes**

1. This protocol supports moderate-throughput sample processing, as multiple whole brains can be processed in parallel within the same container. Up to five whole mouse brains per 20 mL vial have been tested throughout the clearing and refractive index matching steps without compromising tissue integrity, optical clarity, or fluorescence preservation. All samples should be fully submerged and gently agitated to ensure uniform solvent exchange.

2. This protocol may be paused at any 100% methanol dehydration step. For extended pauses, methanol should be replaced weekly to prevent water uptake and degradation of clearing efficiency.

3. Throughout the protocol, gentle agitation is recommended during solvent incubations to facilitate uniform solvent exchange and even tissue processing. Agitation does not need to be vigorous; slow rocking or nutation sufficient to visibly move the solution around the tissue is adequate. Excessive agitation is unnecessary and may increase the risk of mechanical stress or sample damage.

4. Prolonged exposure to DCM should be avoided, as excessive solvent exposure can reduce endogenous fluorescent protein signal intensity. Likewise, storage in DBE should not exceed one week.

5. Regulatory approval or special institutional permission may be required for the use of DCM, depending on local safety and environmental regulations.

6. Scintillation vials may be reused provided they are dedicated to a single solvent type. Vials using methanol should be reserved exclusively for methanol steps, while vials used for DCM or DBE should be used only for steps containing the corresponding solvent to avoid cross-contamination.

7. Solvent-compatible metal or glass tools may be used to transfer brains between containers; all tools should be verified as solvent-safe before use. The use of tissue forceps or blunt, smooth-tipped tweezers is recommended to minimize the risk of compressive damage, surface indentations, or groove formation in the tissue.

8. All solvents should be stored in tightly sealed, solvent-compatible containers at room temperature and protected from prolonged exposure to air to minimize evaporation. Working solutions should be prepared using freshly opened or well-sealed stock solvents, as gradual solvent loss over time can reduce effective concentration and compromise dehydration and delipidation efficiency. DBE and ECi should likewise be stored in airtight containers and protected from light.

9. ECi is used as the final refractive index matching and imaging medium in this protocol. Use of a non-aqueous clearing protocol avoids the need for high-cost aqueous refractive index matching solutions, which can be advantageous for large-volume imaging setups.

**Troubleshooting**

**Problem 1:** Incomplete clearing or residual opacity in the brain.

Possible causes: Incomplete dehydration, insufficient agitation, or use of aged solvent solutions. Volatile organic solvents such as methanol and DCM can evaporate over time, reducing effective solvent concentration and clearing efficiency.

Solutions: Prepare fresh methanol and DCM solutions as recommended and ensure containers are tightly sealed between uses to minimize evaporation. Fully exchange solvents at each step and maintain continuous gentle agitation throughout dehydration and clearing to promote uniform solvent penetration.

**Problem 2:** Visible blood vessels in cleared samples.

Possible cause: Ineffective transcardial perfusion prior to fixation.

Solution: Increase the volume of PBS used during perfusion to improve blood removal before fixation.

**Problem 3:** High background fluorescence.

Possible causes: Suboptimal fixation conditions or impurities in the fixative solution.

Solutions: Use freshly prepared, filtered 4% PFA in PBS for fixation. Filtration of the fixative solution improves signal-to-noise ratio and reduces background fluorescence.

**Problem 4:** Deformed whole-brain samples.

Possible cause: Mechanical distortion during brain extraction, particularly stretching of the cortex from the cerebellum during dissection.

Solution: Perform careful and controlled dissection to minimize mechanical stress and preserve native tissue morphology.

**Problem 5:** Persistent air bubbles within cleared samples (Figure 3).

Possible causes: Trapped air introduced during solution changes or incomplete equilibration in the final imaging medium.

Solutions: Allow samples to equilibrate fully in ECi prior to imaging and apply gentle agitation to facilitate bubble release. Brief incubation at elevated temperature may accelerate bubble dissipation; however, samples should be monitored closely to avoid prolonged exposure.
